# Evaluating Optical Properties of Mixed-Phase 2D MoSe_2_/Poly(vinyl alcohol) Nanocomposite Film

**DOI:** 10.3390/ma17174178

**Published:** 2024-08-23

**Authors:** Suman Chhetri, Anh Tuan Nguyen, Nicolas Gaillard, Woochul Lee

**Affiliations:** 1 Department of Mechanical Engineering, University of Hawaii at Manoa, Honolulu, HI 96822, USA; chhetri@hawaii.edu (S.C.); anhtuann@hawaii.edu (A.T.N.); 2 Hawaii Natural Energy Institute, University of Hawaii at Manoa, Honolulu, HI 96822, USA; ngaillar@hawaii.edu

**Keywords:** MoSe_2_ nanosheet, nanocomposite film, optical properties, atomic force microscopy

## Abstract

Highly solar light-absorbing poly(vinyl alcohol) (PVA) nanocomposite films have garnered wide attention in fields such as flexible optoelectronics, solar energy harvesting, and photothermal therapy. However, fabricating PVA nanocomposite films with a broad spectrum of solar absorption using cost-effective and non-toxic nanofillers remains challenging. Herein, nanocomposite films of PVA incorporating various concentrations of mixed-phase 2D MoSe_2_ nanosheets (i.e., a combination of the 2H and 1T phase) were prepared using a solution casting technique. Scanning electron microscopy (SEM) shows homogenous dispersion of MoSe_2_ nanosheets in the PVA matrix even at higher concentrations, while atomic force microscopy (AFM) reveals increasing surface roughness with increasing MoSe_2_ content, reaching a plateau after 20 wt%. With the increase in the concentration of MoSe_2_, the nanocomposite films exhibit interesting light absorption characteristics reaching their highest absorption (average 94.9%) at 40 wt% MoSe_2_. The incorporated mixed-phase MoSe_2_ nanosheets induce a significant change in the energy levels of the PVA matrix, which is reflected in the reduced optical band gap energy (2.63 eV) at 40 wt% MoSe_2_ against pure PVA (5.28 eV). The excellent light absorption of PVA nanocomposite films across the entire range from 250 nm to 2500 nm is attributed to the thin 2D structure of MoSe_2_ and the presence of its mixed phase.

## 1. Introduction

The development of cost-effective materials with an ultra-high light absorption capacity across a wide wavelength range is crucial for emerging flexible optoelectronic devices, photonics, and solar cells and for realizing a plethora of other applications [1,2,3,4,5]. Polymers are considered an ideal framework, particularly for designing a lightweight, flexible component, and are often employed as a transparent optical system [5]. Promisingly, the fabrication of organic–inorganic nanocomposites has been an efficient route to achieve the requisite properties that cannot be fulfilled by the polymer alone, crucial for protective coatings in optoelectronic devices, highly refractive index films, optical wave guides, etc. [6]. The atomic-level interaction between organic and inorganic components in hybrid materials has been recognized as a factor responsible for the emergence of functional properties. The synergy in interaction at the nano level is often driven by the compatibility between the nanofiller and the polymer along with the quality of nanoparticle dispersion. A polar polymer like poly(vinyl alcohol) (PVA), with its high dielectric constant, is considered an ideal host for promoting nanofiller stability. This stability enables uniform dispersion, resulting in a homogeneous nanocomposite system [7,8,9,10]. The presence of polar groups in the PVA matrix can efficiently inhibit the nanoparticles’ aggregation via coordination with charges or groups present on the surface of the nanofiller, making it a desirable host for composite systems [9,10].

The incorporation of nanoparticles into a semi-crystalline and water-soluble PVA matrix to form nanocomposites demonstrated changes in optical properties, where the added particles induce a significant change in the electronic band structure of the polymer [3,11]. Metal nanoparticles or various metal oxides such as CuO, ZnO, CeO_2_, and Bi_2_O_3_ have been added to PVA in different concentrations to tune its optical properties [1,3,4,6,12,13]. Biosynthesized silver nanoparticle-encapsulated PVA films demonstrated a decreasing optical energy band gap with increasing silver concentration, manifesting the nanoparticle-induced alteration in the electronic structure of PVA composites [8]. A similar trend in band gap has been evident in PVA nanocomposite films containing various types of nanoparticles such as CuO, ZnO, Bi_2_O_3_, MnCl_2_, and so forth [2,3,6,13,14]. It is substantiated that an electronic interaction between the added nanofiller and the polymer matrix leads to enhanced absorption intensity [4,5]. This is attributed to the fact that the embedded nanoparticles can create numerous localized energy levels, referred to as trapping sites [4]. Lately, linear and non-linear optical properties of graphene-like transition metal dichalcogenides (TMDs)-based PVA nanocomposite films have been reported [11,15]. The 2D graphene-like layered TMD materials with a chemical formula of MX_2_ (M is a transition metal and X is a chalcogen) have exhibited versatile electrical and optical properties and could therefore be considered promising as charge-trapping elements for various applications [11,16,17,18]. Due to quantum-mechanical confinement, single- and few-layered MX_2_ materials show unique physical and chemical properties against their bulk counterparts. These properties arise from their enormous surface-to-volume ratios, rich surface-active sites, and unique band structures [16].

Among the TMDs, mono- or multi-layered molybdenum diselenide (MoSe_2_) with a graphene-like structure has been preferred over molybdenum disulfide (MoS_2_) because of its structural stability and mechanical flexibility [11,16]. The intrinsic metallic nature of selenium endows MoSe_2_ with higher electrical conductivity, which makes it a potential candidate when compared to other TMDs [11]. It possesses two phases, thermodynamically unstable 1T-metallic and 2H-semiconducting phases, and has a tunable band gap, which is ~1.1 eV in bulk material and ~1.58 eV in monolayered form [19]. The literature has documented an excellent visible light absorption property of 2D MoSe_2_ [16,20]. However, broad-spectrum light absorption abilities are desirable for materials to be used in various solar harvesting applications. The 1T-phase of MoSe_2_ has been considered to have strong absorption in the NIR region akin to MoS_2_ [16,21]. Therefore, the synthesis of a mixed phase of MoSe_2_ (i.e., a combination of the 2H and 1T phases) and its incorporation into a polymer matrix offer a potential route to develop nanocomposites with broad solar absorption attributes. The precise modulation of defects and interfaces is expected to be achieved from controllable phases of MoSe_2_, which can lead to efficient light absorption. In comparison to the myriad of other investigations associated with MoSe_2_, the impact of mixed phases of MoSe_2_ on the light absorption properties of composite films remains unexplored. The hydrophilic nature and low-level toxicity of MoSe_2_ make it well-suited for preparing nanocomposites for various applications [22]. In this study, 2D MoSe_2_/PVA nanocomposite films are prepared using different weight percentages of MoSe_2_ nanosheets. Due to its non-toxicity and biodegradable characteristics, PVA was selected as a host material for the semiconducting MoSe_2_. The impact of embedded mixed-phase 2D MoSe_2_ on the light absorption properties of PVA composite films was investigated along with the MoSe_2_-induced change in the optical band gap energy. Imaging techniques such as scanning electron microscopy (SEM) and atomic force microscopy (AFM) were employed to examine the degree of MoSe_2_ dispersion in the PVA matrix.

## 2. Materials

Poly(vinyl) alcohol Mw 89,000–98,000 (99% hydrolyzed), ammonium molybdate tetrahydrate, selenium dioxide, and hydrazine monohydrate N_2_H_4_, 64–65% reagent grade 98%, were purchased from Sigma Aldrich (St. Louis, MO, USA). All the chemicals were used as received.

### 2.1. Synthesis of Graphene-like 2D MoSe_2_

A hydrothermal route was employed to prepare MoSe_2_ nanosheets, which were prepared at two different temperatures, 180 °C and 200 °C, maintaining the same duration (24 h). Briefly, a solution containing 777 mg (7 mmol) of SeO_2_ in 20 mL of water was prepared to which 15 mL of hydrazine was slowly added and left stirring for 2 h. Another solution containing 618 mg of ammonium molybdate (0.5 mmol) was prepared separately. The solution containing selenium was then added dropwise to ammonium molybdate while stirring and left to stir for 2 h at room temperature. The homogenous solution was then transferred to a 100 mL Teflon-lined autoclave (Huanyu, China) and kept at 180 °C and 200 °C for 24 h. After cooling the autoclave to room temperature, the product was taken out and filtered with DI water and ethanol. After vacuum drying for 12 h at 60 °C, graphene-like 2D MoSe_2_ was achieved. 

### 2.2. Preparation of PVA/2D MoSe_2_ Nanocomposite Film

To prepare pure PVA film, 1 gm of PVA was dissolved in DI water while stirring in an oil bath for 2 h at 80 °C. After stirring, the homogeneous solution was allowed to cool, then cast onto a clean Petri dish and left to dry at room temperature. PVA/MoSe_2_ nanocomposite films were prepared with different weight percentages of MoSe_2_ (from 1 wt% to 50 wt%). At first, the required amount of MoSe_2_ was dispersed in DI water using bath sonication for 40 min, which was then added to the PVA solution and then left stirring for 1 h. The homogeneous PVA/MoSe_2_ solution that formed after an hour of stirring was then cast onto a petri dish and left for water evaporation and drying at room temperature. After the complete removal of water, the composite film of a thickness of 0.025 ± 0.005 mm was peeled off from the petri dish and kept for further characterization. 

### 2.3. Characterization Technique

The morphology of MoSe_2_ and MoSe_2_/PVA nanocomposite films was observed by SEM Hitachi S-4800 (Hitachi, Ltd., Hitachinaka, Japan). Specimens were mounted with conductive carbon tape on aluminum stubs, sputter-coated with palladium in a Hummer 6.2 sputter coater, and viewed at an accelerating voltage of 2.0 kV. The total transmittance and reflectance of pristine PVA and PVA/MoSe_2_ nanocomposite samples were measured with an integrating sphere in the wavelength range of 250–2500 nm using a PerkinElmer LAMBDA 750 S UV/Vis/NIR spectrophotometer (PerkinElmer, Waltham, MA, USA). The topography of the pristine PVA and PVA/MoSe_2_ nanocomposite films was characterized using atomic force microscopy (AFM) (NX10, The Park System, Suwon, Republic of Korea). The non-contact mode was performed by employing an NCHR (spring constant = 42 N/m) probe (Nanosensors, Neuchatel, Switzerland); the surface roughness (expressed by root mean square) of the films was then calculated from the topography.

## 3. Results and Discussion

**Figure 1:**
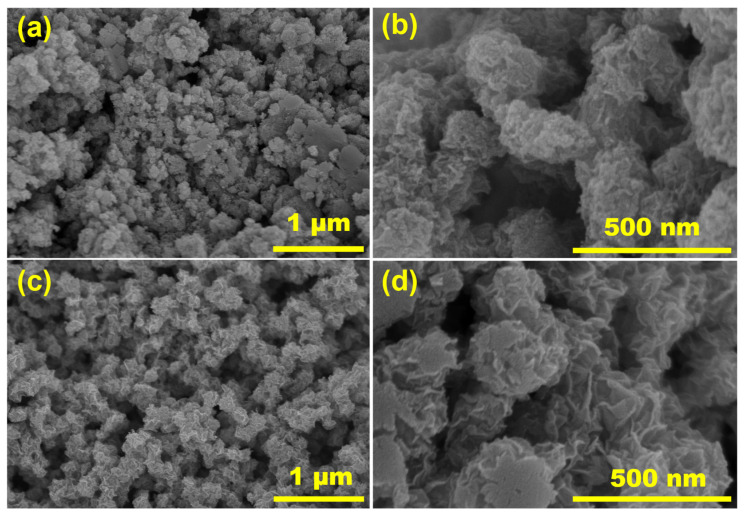
SEM micrographs of graphene-like 2D MoSe_2_ with different magnification. (**a**,**b**) Microstructure of MoSe_2_ nanoparticles prepared at 180 °C. (**c**,**d**) SEM images of MoSe_2_ nanoparticles prepared at 200 °C through hydrothermal route for 24 h.

**Figure 2:**
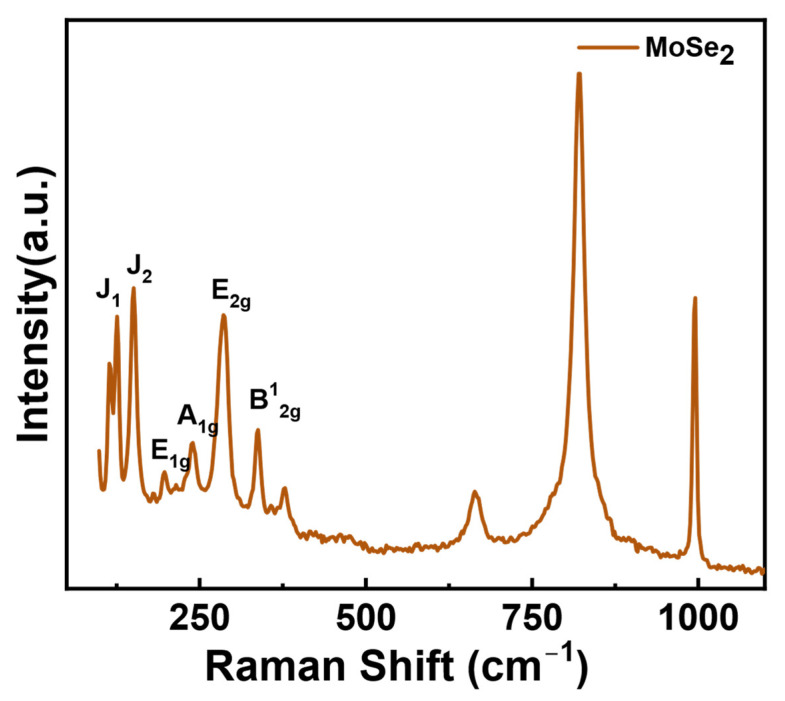
A Raman spectrum of the MoSe_2_ nanoflower.

SEM was employed to study the morphological characteristics of the prepared MoSe_2_ nanosheets at two different temperatures (180 °C and 200 °C) as depicted in Figure 1. As observed in the micrograph, the MoSe_2_ prepared at 180 °C is not a perfect assembly of a MoSe_2_ nanosheet (Figure 1a,b). The nanosheets form a more compact structure, where the individual sheets are not fully dispersed and extended. Rather, the petals appear to close inward, forming a microstructure that does not resemble a perfect flower. On the other hand, the MoSe_2_ prepared at 200 °C forms a flower-like assembly of nanosheets, where the crumpled and curled sheets of graphene-like MoSe_2_ are arranged in a 3D structure (Figure 1c,d). The nanosheets are more expanded and assembled in a well-defined morphology. An atomic force microscopy study was used to estimate the thickness of the MoSe_2_ nanosheets prepared at 200 °C, which was found to be 14 nm. The temperature-governed morphology of MoSe_2_ showed an interesting pattern of nanosheet arrangement in a flower-like structure. As the microstructure of MoSe_2_ achieved at 200 °C is more precise and definitive, we choose it for further characterization and nanocomposite film fabrication. The Raman spectrum of the as-prepared MoSe_2_ exhibited vibration modes corresponding to both the 1T phase as well as the 2H phase (Figure 2). The J_1_ mode at 125.5 cm^−1^ and the J_2_ mode at 150.4 cm^−1^ were ascribed to the 1T phase, while E_1g_ at 197 cm^−1^, A_1g_ at 238.7 cm^−1^, E_2g_ at 286 cm^−1^, and B^1^_2g_ at 337.7 cm^−1^ were accounted to the 2H phase. The presence of the B^1^_2g_ mode is indicative of an ultrathin layered structure [16]. The presence of two types of vibration modes confirmed the integration of 1T and 2H phases of as-prepared MoSe_2_.

**Figure 3:**
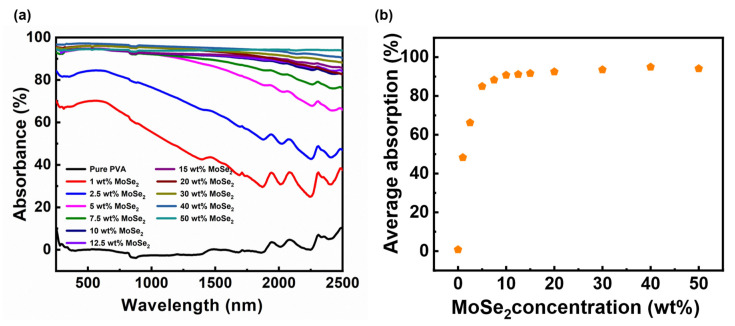
Light absorption properties of PVA/MoSe_2_ composite films. (**a**) UV-Vis NIR absorbance spectra of pure PVA and PVA composite films containing different mass concentrations of MoSe_2_ plotted against wavelength. (**b**) Average absorption of PVA/MoSe_2_ composite films with different MoSe_2_ concentrations.

Figure 3a presents the light absorption spectra of pure PVA and PVA nanocomposite films containing different weight percentages of MoSe_2_. The average absorption versus MoSe2 concentration is displayed in Figure 3b, where the average absorption is calculated over the studied wavelength range of 250 to 2500 nm. As expected, pure PVA film is transparent to light within the studied spectral range of 250–2500 nm. The spectra of the PVA/MoSe_2_ nanocomposite films revealed a progressive increase in light absorption with increasing MoSe_2_ concentration reaching a maximum at 40 wt%. PVA composite films exhibit a significant and abrupt enhancement in light absorption within the UV-Vis region at up to 10 wt% MoSe_2_, while composites beyond this concentration demonstrate moderate improvements. With increasing MoSe_2_ concentration, the absorption in the NIR region gradually straightens up, achieving a nearly constant value at 50 wt% MoSe_2_. The gap in absorption enhancement between consecutive weight percentages in both the UV-Vis and NIR region is wide (up to 10 wt%), after which the gap gradually narrows, reaching maximum absorption at 40 wt% MoSe_2_ (94.9%). The calculated average value is comparable to the absorption exhibited by PVA nanocomposite containing 45 wt% modified Ti_2_O_3_ nanoparticles [23]. The PVA composite film at 50 wt % of MoSe_2_ showed diminished absorption particularly in the UV-Vis region compared to the composite film containing 40 wt% MoSe_2_. The average absorption was found to be 94% at 50 wt% MoSe_2_.

The transmittance spectra of pure PVA and PVA/MoSe_2_ composite films are presented in Figure S1a. Pure PVA exhibits transmittance in the range of 91–92% within the spectral range being studied, and with the addition of MoSe_2_, the transmittance of PVA films diminished. At the higher weight percentage of MoSe_2_ (after 30 wt%), its transmittance remained below 0.9%. Notably, the composite film becomes almost opaque after 2.5 wt% MoSe_2_ within the UV-Vis region, while in the NIR region, the transparency gradually diminishes with increasing MoSe_2_ concentration reaching complete opaqueness at 30 wt%. The reflectance spectra of the PVA/MoSe_2_ nanocomposite films are shown in Figure S1b. The higher reflectance observed in the NIR region at a lower weight percentage of (1 wt % and 2.5 wt%) MoSe_2_ can be explained by the sea–island structure of the PVA composite films [24,25]. The PVA matrix and non-continuous MoSe_2_ distribution are considered as sea and island, respectively. At a lower weight percentage of MoSe_2_, the small islands indicating MoSe_2_ are disconnected from the PVA matrix forming boundaries and interfaces. It is considered that poor connection between those island structures at lower weight percentages of MoSe_2_ causes an interfacial impedance mismatch, leading to higher reflectance. As the weight percentage of MoSe_2_ increases, the population of islands in the PVA matrix also increases and becomes more connected, which helps in accomplishing impedance matching and diminishes the reflection of the incident beam at the composite–air interface.

The significant enhancement in absorption is attributed to the presence of 1T-phase and 2H-phase MoSe_2_, which is in agreement with a previous finding suggesting the major contribution of the 1T phase particularly in the NIR region [16]. The 1T-MoSe_2_ phase with an approximate band gap of 1.1 eV exhibits metallic features, while 2H-MoSe_2_ behaves like a typical semiconductor. Further, the boost in absorption can also be accounted for by the well-expanded ultrathin graphene-like MoSe_2_ layered structure, which is considered beneficial for internal reflections. The flower-like structure of MoSe_2_ can increase the roughness of PVA films, further improving light absorption. As documented in previous studies [26], the numerous defects present in MoSe_2_ could also contribute to the strong light absorption of PVA nanocomposite films.

**Figure 4:**
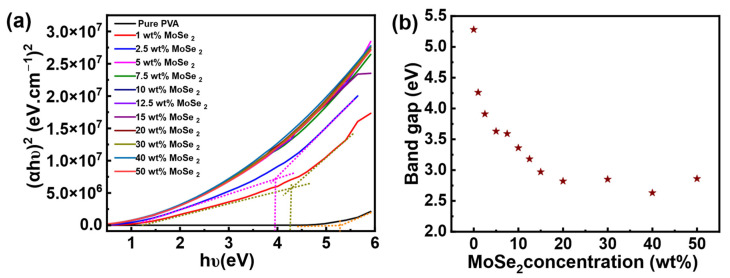
Band gap energy estimation of PVA and PVA/MoSe_2_ composite films. (**a**) Plot of (*αhυ*)^2^ against photon energy *hυ* for pure PVA and PVA composite films containing different concentrations of MoSe_2_. For clarity of presentation, calculation of optical band gap using baseline approach is represented only for pure PVA, and PVA nanocomposite films containing 1 and 2 wt% MoSe_2_. (**b**) Band gap of PVA composite films corresponding to different concentrations of MoSe_2_.

UV-Vis-NIR spectroscopy in combination with the Tauc method was used to estimate the optical energy band gap of PVA nanocomposite films containing different concentrations of MoSe_2_ using Tauc’s relation [8,27,28]:

(1) $${\left(\alpha h\vartheta \right)}^{\frac{1}{n}}=B\left(h\vartheta -E_{g}\right)$$

where *α* is the absorption coefficient, *h* is Planck’s constant, *B* is Tauc’s constant related to transition probability, *υ* is the light frequency, and *E_g_* is the optical energy band gap. The exponent *n* relates to the type of electronic transitions, with values of ½ for direct transitions and 2 for indirect transitions. Absorbance, *A*, is normalized to the thickness of the nanocomposite films to calculate the absorption coefficient *α* using the following equation:

(2) $$\alpha =\frac{2.303\;A}{t}$$

where *t* is the thickness of the nanocomposite sample. To reduce potential errors in estimating optical band gap values, a baseline approach was employed. According to the method described by Tauc, the linear portion of the plot ((*αhυ*)^2^ versus *hυ*) is extrapolated to the photon energy axis (where (*αhν*)^2^ equals zero) to estimate the band gap value. However, this approach can lead to inaccuracies in estimating band gap values if it does not account for low-energy defect absorption. Specifically, the linear segment of the curve, predominantly in the high-energy region of Tauc’s plot, characterizes the fundamental absorption of the materials, while the non-linear segment relates to absorption associated with defect states [29,30]. If the baseline corresponding to the low-energy absorption state is not considered, the estimation can be skewed, leading to a misunderstanding of the material’s optical properties [30,31]. To address this, the method applied in this work accounts for low energy defect absorption by drawing a baseline that intersects with the linear fit of the main absorption. This allows for a vertical line to be dropped to the energy axis, where the optical band gap is determined [32], as depicted in Figure 4a. The values of band gap energy for all the studied composite films are displayed in Figure 4b. The measured band gap energy value for pure PVA is 5.28 eV, and the value decreases with an increase in the mass concentration of MoSe_2_ and reaches 2.63 eV at 40 wt%. It is recognized that the inorganic filler alters the electronic structure of PVA by creating localized energy states between the conduction and valence bands [8]. The significant decrease in the band gap energy of the composite films can be ascribed to the fact that the embedded MoSe_2_ can form localized electronic states that behave as trapping and recombination centers leading to the modification in the optical band gap. The excitation of electrons from the valence band to the conduction band progresses through the localized levels of the nanoparticle clusters, leading to a decrease in band gap energy. This decrease in band gap energy also suggests a change in the primary structure of PVA composites induced by the embedded MoSe_2_ [33].

**Figure 5:**
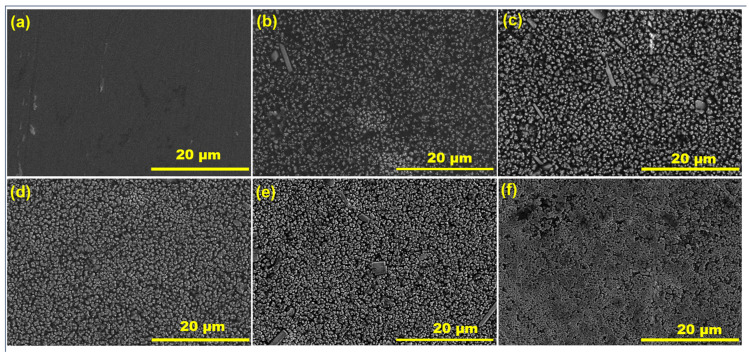
Microstructure of PVA and PVA/MoSe_2_ composite films containing different concentrations of MoSe_2_. (**a**) SEM micrograph of pure PVA, (**b**) SEM image of PVA/MoSe_2_ film containing 5 wt% MoSe_2_, (**c**) 10 wt% PVA/MoSe_2_, (**d**) 20 wt% PVA/MoSe_2_, (**e**) 30 wt% PVA/MoSe_2_, and (**f**) 40 wt% PVA/MoSe_2_.

To elucidate the extent of dispersion of MoSe_2_ nanoparticles within the PVA matrix, morphological imaging was acquired using SEM, and the micrographs of pure PVA and composite films are depicted in Figure 5a–f. The pure PVA film exhibits a smooth surface without any evident heterogeneity (Figure 5a). The morphologies of PVA composite films with different concentrations of MoSe_2_ showed increasing continuity with increasing MoSe_2_ mass. At 5 wt%, as seen in Figure 5b, though the nanoparticles are homogenous, they are not yet interconnected and do not continuously cover the entire space within the PVA matrix. The nanoparticles appear closer at 10 wt% MoSe_2_ (Figure 5c) and the covering becomes more apparent. As can be seen at higher MoSe_2_ concentrations (20–40 wt%) in the composite films, the number of nanoparticles per unit area increases significantly, leading to a tight arrangement of MoSe_2_ within the PVA matrix (Figure 5d,e). The nanoparticles are inter-connected and homogenously fill the space, forming a perfect covering layer on the PVA substrate. It is interesting to observe that even though nanoparticles within the PVA matrix are compact and continuously arranged, there is no evident agglomeration and overlapping of MoSe_2,_ indicating excellent compatibility. Despite the large mass concentration of MoSe_2_ in the PVA matrix, the nanoparticles are still homogenous in distribution. Also, there are no visible voids at the interface between the PVA matrix and MoSe_2,_ indicating strong interfacial bonding. The intriguing arrangement of MoSe_2_, forming a continuous layer encompassing the entire PVA matrix, likely impacted the light absorption attributes of the composite film.

**Figure 6:**
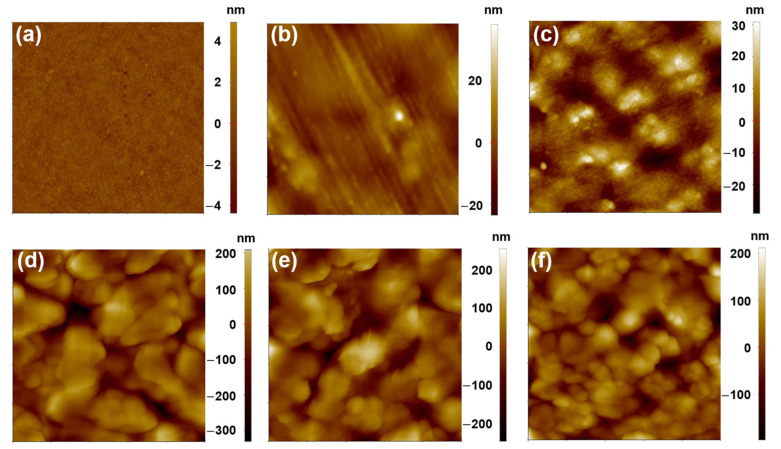
AFM topography images of the pristine PVA and PVA/MoSe_2_ composite films containing different concentrations of MoSe_2_. (**a**) Pure PVA, (**b**) 1 wt% PVA/MoSe_2_ film, (**c**) 5 wt% PVA/MoSe_2_ film, (**d**) 20 wt% PVA/MoSe_2_ film, (**e**) 30 wt% PVA/MoSe_2_ film, and (**f**) 40 wt% PVA/MoSe_2_ film. The scan size in (**a**–**f**) is 5 × 5 µm^2^.

To further confirm the homogeneity and examine the surface roughness of the MoSe_2_-embedded PVA composite films, AFM in a non-contact mode was used. Figure 6a–f show the morphological features of pure PVA and PVA nanocomposite films. Pure PVA film does not show any unique features under AFM studies. Two distinct regions can be observed in the topography images of the PVA nanocomposite films: the bright, elevated features are associated with MoSe_2_ nanoparticles, and the brown base corresponds to the PVA matrix. The microstructure features resemble jackfruit pods, and it appears that the pods are assembled around the dark brownish pit.. As seen in Figure 6b–f, an evident bright assembly of the pods is observed in MoSe_2_-incorporated PVA, which is absent in the pure PVA film (Figure 6a). Distinctly different features can be observed in the composite films at lower mass concentrations and at higher concentrations of MoSe_2_. At lower concentrations of MoSe_2_ (up to 10 wt%), the bright spots that correspond to MoSe_2_-rich zones are isolated and are not interconnected (Figure 6c and Figure S2a). On the other hand, at higher concentrations of MoSe_2_ (above 10 wt%), the bright spots are interconnected, assuming a continuous structure (Figure 6d–f and Figure S2b). The homogenous layer that emerged from the interconnected MoSe_2_ particles is more striking at 40 wt% PVA composite film, suggesting fine dispersion even at such a high mass concentration. The topographical features observed in the AFM study perfectly align with the SEM micrographs of the PVA nanocomposite films. At lower mass concentrations, MoSe_2_ nanoparticles are not interconnected, while with increasing concentration, the particles tend to start interconnecting, forming a continuous structure, which also corroborates the calculated surface roughness values expressed in root mean square (RMS) in Figure S3. It is suggested that MoSe_2_ is not continuous in the composite film up to 10 wt%. Beyond this composition, the nanoparticles form a continuous structure, leading to a significant change in surface roughness. The surface roughness of the PVA/MoSe_2_ composite films increases upon increasing the mass of the MoSe_2_ in the PVA matrix reaching a maximum at 20 wt% and exhibiting a slight decrease above this composition. The increased surface roughness of the PVA composite films with increasing MoSe_2_ concentration can be linked to the enhanced light absorption resulting from the enhanced diffuse scattering and path length.

## 4. Conclusions

In conclusion, 2D graphene-like mixed phases of MoSe_2_ (2H-MoSe_2_/1T-MoSe_2_) were prepared following a one-step hydrothermal method. The optical properties of PVA nanocomposite films containing different mass concentrations of MoSe_2_ were studied, revealing the concentration dependency on these properties. Due to the mixed phases of MoSe_2_ with abundant defects, PVA/MoSe_2_ nanocomposite films exhibit intriguing absorption characteristics as MoSe_2_ concentration increases. At 40 wt% of MoSe_2_, the composite film shows an average absorption value of 94.9%. The incorporation of mixed-phase MoSe_2_ nanosheets alters energy levels in PVA composite films (2.63 eV at 40 wt% MoSe_2_), resulting in diminished optical band gap energy compared to the pure PVA matrix (5.28 eV). The microstructure of PVA composite films with different mass concentrations of MoSe_2_ exhibited increasing continuity with increasing MoSe_2_ mass without evident agglomeration. The seamless arrangement of MoSe_2_ nanosheets forming a forest of particles covering the entire PVA matrix potentially impacted the light absorption behavior of composite films. The change in surface topography was quantitatively determined by calculating surface roughness from AFM studies. The increasing surface roughness with MoSe_2_ mass concentration corroborated the morphological features of the PVA composite films observed in SEM studies. The excellent light absorption across a wide spectrum makes this developed composite film a potential candidate for emerging flexible optoelectronic devices, photonics, solar cells, and related fields.

## Data Availability

The data supporting the conclusion of this work are available from the corresponding author on reasonable request.

## Supplementary Materials

The following supporting information can be downloaded at https://www.mdpi.com/article/10.3390/ma17174178/s1, Figure S1: UV-Vis-NIR spectra of pure PVA and PVA/MoSe_2_ composite films. (a) Transmittance spectra of pure PVA and PVA composite films containing different concentrations of MoSe_2_. (b) Reflectance spectra of pure PVA and PVA composite films containing different concentrations of MoSe_2_; Figure S2: AFM topography images of PVA composite films: (a) PVA film containing 10 wt% MoSe_2_ and (b) PVA film containing 15 wt% MoSe_2_; Figure S3: Variation in surface roughness expressed in root mean square of pristine PVA and PVA composite films with respect to MoSe_2_ concentration.
