# Supplementary material for: Evaluating Optical Properties of Mixed-Phase 2D MoSe2/Poly(vinyl alcohol) Nanocomposite Film
Source: Materials (Basel). 2024 Aug 23;17(17):4178. doi: 10.3390/ma17174178 (PMC11395827; doi:10.3390/ma17174178)
Supplement: Supplementary file 1 [file materials-17-04178-s001.zip › materials-3125358-supplementary.pdf]

Evaluating Optical Properties of Mixed-Phase 2D $MoSe_2$/Poly(Vinyl Alcohol) Nanocomposite Film

Suman Chhetri[1], Anh Tuan Nguyen[1], Nicolas Gaillard[2], Woochul Lee[1, *]

[1]Department of Mechanical Engineering, University of Hawaii at Manoa, Honolulu, HI 96822, USA

[2]Hawaii Natural Energy Institute, University of Hawaii at Manoa, Honolulu, HI 96822, USA

Corresponding author email: **woochull@hawaii.edu**

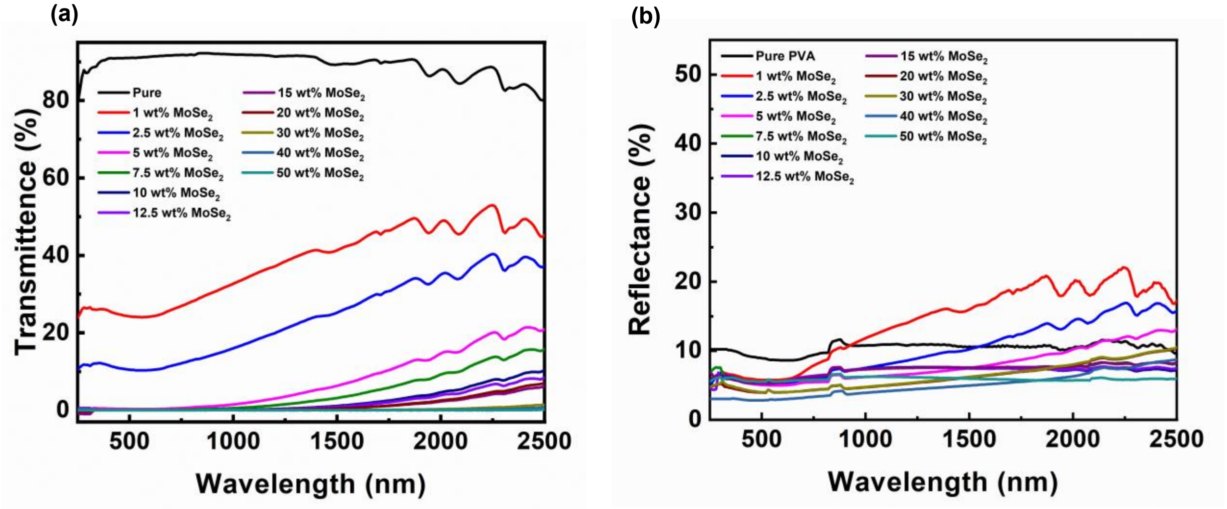

Figure S1 UV-Vis-NIR spectra of pure PVA and PVA/$MoSe_2$ composite films. (a) Transmittance spectra of pure PVA and PVA composite films containing different concentrations of $MoSe_2$. (b) Reflectance spectra of pure PVA and PVA composite films containing different concentrations of $MoSe_2$.

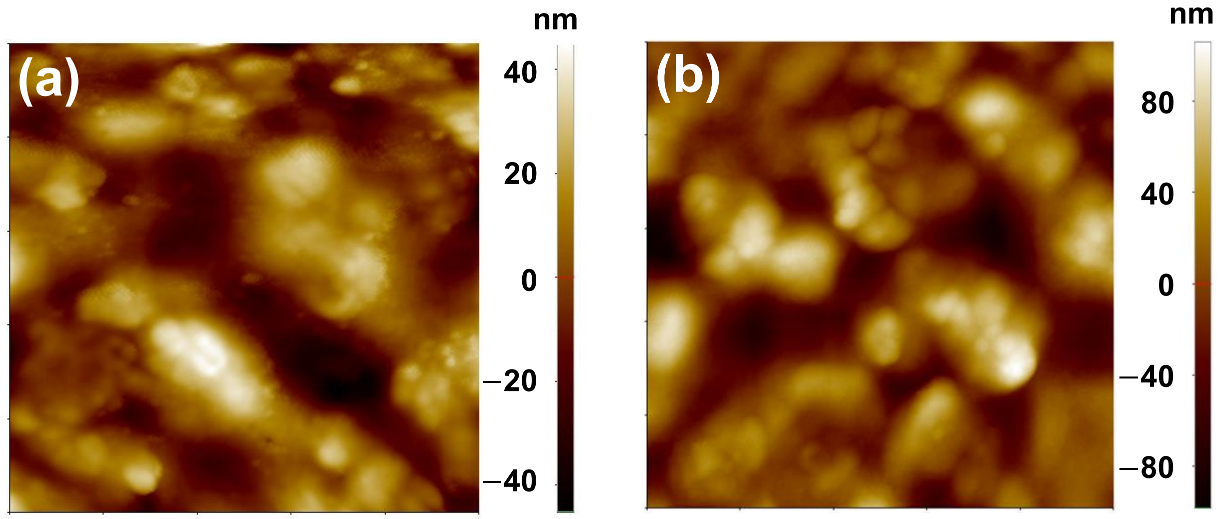

Figure S2 AFM topography images of PVA composite films (a) PVA film containing 10 wt% $MoSe_2$ and (b) PVA film containing 15 wt% $MoSe_2$

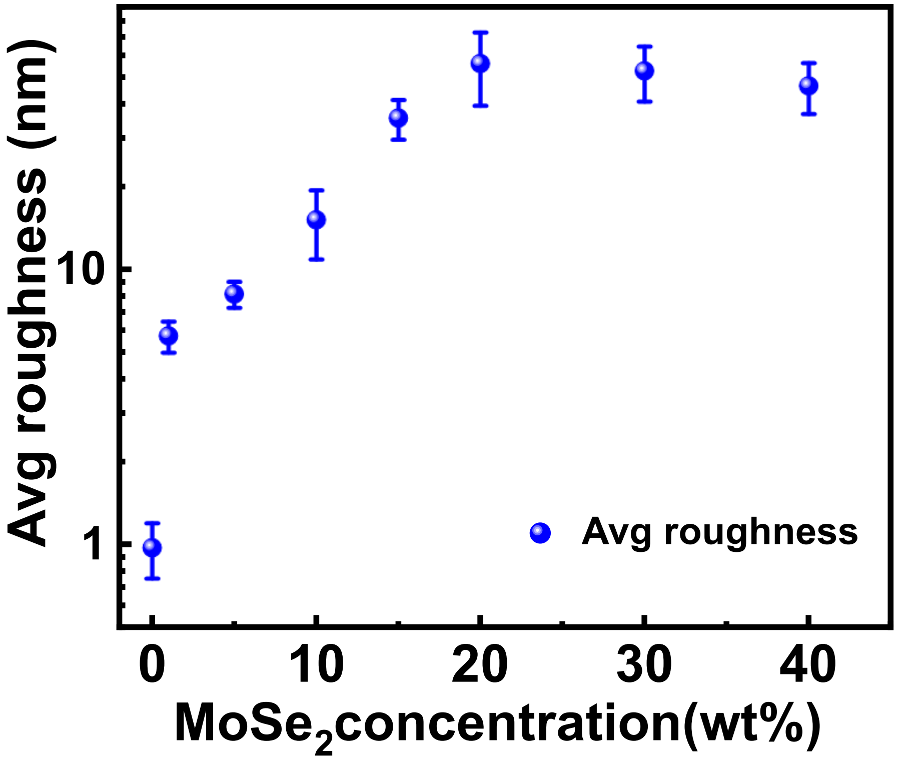

Figure S3 Variation in surface roughness expressed in root mean square of pristine PVA and PVA composite films with respect to $MoSe_2$ concentration.
